# Individual Differences in Belief in Fake News about Election Fraud after the 2020 U.S. Election

**DOI:** 10.3390/bs11120175

**Published:** 2021-12-10

**Authors:** Dustin P. Calvillo, Abraham M. Rutchick, Ryan J. B. Garcia

**Affiliations:** 1 Psychology Department, California State University, San Marcos, San Marcos, CA 92096, USA; 2 Psychology Department, California State University, Northridge, Los Angeles, CA 91330, USA; abraham.rutchick@csun.edu; 3 Defense Resources Management Institute, Naval Postgraduate School, Monterey, CA 93943, USA; ryan.garcia@nps.edu

**Keywords:** fake news, elections, misinformation, conspiracy beliefs, political ideology

## Abstract

Fake news is a serious problem because it misinforms people about important issues. The present study examined belief in false headlines about election fraud after the 2020 U.S. presidential election. Belief in election fraud had dangerous consequences, including the deadly insurrection at the U.S. Capitol in January 2021. In the present study, participants rated the truthfulness of true and false headlines about the election, and then completed individual difference measures eight days after the election. Participants with more conservative ideology, greater presidential approval of the outgoing president, greater endorsement of general conspiracy narratives and poorer cognitive reflection demonstrated greater belief in false headlines about election fraud. Additionally, consuming more politically conservative election news was associated with greater belief in false headlines. Identifying the factors related to susceptibility to false claims of election fraud offers a path toward countering the influence of these claims by tailoring interventions aimed at decreasing belief in misinformation and decreasing conspiracy beliefs to those most susceptible.

## 1. Introduction

Fake news—the presentation of false or misleading information as if it were legitimate journalism—is rampant. Although fake news is perceived as a serious problem by the public [1], and despite the ongoing attempts of social media platforms to limit its spread, the problem appears to be increasing. The share of social media engagements from unreliable news sites doubled from 2019 to 2020 [2]. Similarly, interactions with deceptive sites have increased on Facebook and Twitter in recent years [3]. The stakes are high, as fake news can misinform people about important issues, such as climate change [4] and COVID-19 [5]. More generally, fake news may threaten the ability of people to agree on what is true [6]. As a recent example, an Ohio nurse testified that the COVID-19 vaccine had caused her to become magnetized, and she attempted to demonstrate this before the Ohio House Health Committee using a hairpin and a (non-magnetic) key, which failed to adhere to her (non-magnetized) body [7]. This extraordinary credence raises the following question: who believes this kind of thing?

Believing in false stories about election fraud has dangerous consequences, particularly when people believe the fraud was the result of a conspiracy. For example, a former police captain pulled a gun on a man he falsely believed was the organizer of an election fraud scheme [8]. Additionally, groups motivated by the election fraud narrative armed themselves and protested outside an election official’s residence [9], while others led a protest near the nation’s capital that led to violence [10]. Furthermore, some election officials and politicians who publicly asserted that no fraud had occurred received threats of violence [11]. Belief in this narrative culminated in the deadly January 6 insurrection at the U.S. Capitol [12]. Thus, falsely perceiving election fraud to have occurred, as with believing other conspiracy narratives, can have negative and even dangerous consequences.

The purpose of this study was to examine factors related to believing fake news about voter fraud in the week after the 2020 U.S. election. Several factors have been identified that relate to belief in false headlines in contexts other than the 2020 U.S. election. The political ideology of participants and the political leaning of headlines affect their perceived accuracy. Participants rate headlines consistent with their political ideology as more accurate than those that are inconsistent with it [13,14,15,16,17,18,19,20,21]. Additionally, political conservatism, approval of President Trump, the perception that COVID-19 was the result of a conspiracy and the perception that the media had exaggerated the threat of COVID-19 were all negatively associated with the ability to discern true or false COVID-19 news [22]. Furthermore, several studies have reported that participants with greater cognitive reflection, defined as the ability to engage in deliberative thinking to reach a correct answer, are better at discerning true from false news [13,16,19,23,24], while others have reported that greater educational attainment is related to better news discernment [19,25]. Finally, news consumption is related to belief in false headlines. The more right-leaning news that participants consume (in particular, Fox News), the poorer their political news discernment [15]. Based on these findings, we examined the roles of political ideology, approval of the outgoing president, trust in mainstream media, cognitive reflection, education, susceptibility to conspiracy narratives and news consumption in the perceived truth of false headlines about election fraud. We believe that understanding individual differences in believing fake news about election fraud allows for the targeting of interventions to those most at risk. 

We had several predictions. We predicted that conservative political ideology, greater approval of the outgoing president and greater endorsement of conspiracy beliefs would be positively associated with greater belief in fake news about election fraud, whereas education, cognitive reflection and trust in news media would be negatively associated with greater belief in fake news about election fraud. We did not make predictions about the relationship between news consumption and belief in fake news. 

## 2. Materials and Methods

### 2.1. Pregistration

Our predictions and planned analyses were preregistered on the Open Science Framework (OSF: https://osf.io/hrcm3/; accessed on 10 November 2020). Our materials and data are also available on the OSF.

### 2.2. Participants

Sample size was determined by a power analysis. We set the smallest bivariate relationship of interest to be *r* = 0.15. According to G*Power [26], we needed 346 participants to have power of 0.80 to detect relationships of this size (with two-tailed α = 0.05). This sample size gave the model with six predictors power of 0.80 to detect an effect size of f^2^ = 0.04, according to G*Power. We preregistered the plan to collect data from 400 participants to account for some participants failing honesty check questions (described below).

Participants were Mechanical Turk workers living in the United States. A total of 401 participants completed the study, and 376 of them passed honesty check questions. Of those 376 participants, 198 of them (52.7%) identified as female, 175 (46.5%) as male and 3 (0.8%) declined to respond to the gender question. Participants ranged in age from 19 to 75 (*Mdn* = 40) years; 221 (58.8%) had a college degree and 155 (41.2%) did not; 183 (48.7%) identified as Democrats, 108 (28.7%) as Republicans and 85 (22.6%) as neither; 201 (55.9%) reported voting for Biden, 117 (31.3%) for Trump and 16 (4.3%) for another candidate, while 33 (8.8%) reported not voting in the 2020 election.

### 2.3. Materials and Procedure

**Figure 1:**
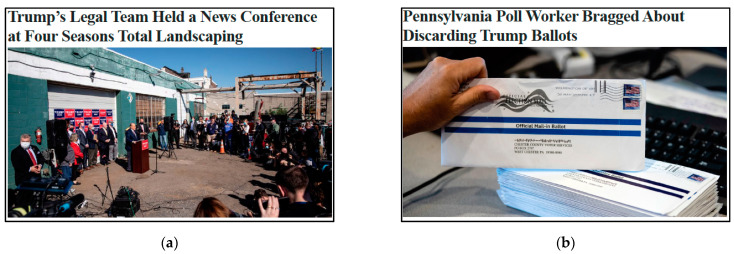
Example of true (**a**) and false (**b**) headlines as they appeared to participants.

After providing informed consent, participants rated the truth of 15 election-related headlines on a scale from 1 (definitely false) to 6 (definitely true). All headlines were fact checked by Snopes.com: 5 of them were determined to be true and 10 false. Of the 10 false headlines, 9 were specifically about irregularities and fraud. These headlines were not specifically about conspiracies of election fraud, but they were consistent with those beliefs. We selected these headlines from the available headlines with truth determinations on Snopes.com in the days after the election. We aimed to gather as many false headlines as we could and ended up with 9. Truth judgments of these 9 headlines were the primary dependent measure. Headlines appeared with a picture to simulate how people encounter these headlines in social media environments. This is common practice in research on fake news [22,23,24], although the inclusion of pictures increases belief in headlines [27]. Example true and false headlines appear in Figure 1. The order of the headlines was randomized for participants. The internal consistency of ratings of these 9 items was good, at α = 0.88.

Participants then completed a 6-item cognitive reflection measure. This set of questions has been used in recent research on fake news [28]. Each item had an intuitive but incorrect response and a correct response that required more deliberation. An example item is, “If you’re running a race and you pass the person in second place, what place are you in?” The intuitive response is first place, and the correct response is second place. Responses were open-ended for all 6 items. We scored the number of correct responses. The internal consistency of ratings of this 6-item measure was acceptable, at α = 0.80, which was slightly greater than previously reported, α = 0.69 [28].

Next, participants completed the 15-item generic conspiracist beliefs scale [29]. The items assess conspiracist ideation without invoking specific conspiracy theories. An example item is, “Certain significant events have been the result of the activity of a small group who secretly manipulate world events.” Participants responded on a scale from 1 (definitely not true) to 5 (*definitely true*) and we took the mean of these ratings. The internal consistency of ratings of this 15-item measure was excellent, at α = 0.95, which is similar to what has been previously reported, α = 0.93 [29].

We created a 5-item measure to examine participants’ trust in mainstream media coverage of the election (adapted from [30]). The stem to each question was, “Mainstream media coverage of the election,” and the five items ended with, “has been fair,” “has been accurate,” “has been unbiased,” “has told the whole story” and “can be trusted.” Participants responded to each item on a scale from 1 (strongly disagree) to 5 (strongly agree) and we took the mean of these ratings. The internal consistency of ratings of this 5-item measure was excellent, at α = 0.97.

Participants then answered demographic questions, including their political party, political ideology on a 1 (extremely liberal) to 7 (extremely conservative) scale, age, gender and education. Participants also stated who they voted for in the election and their level of approval of the outgoing president on a 1 (strongly disapprove) to 4 (strongly approve) scale, and they provided information about their news consumption. Specifically, they selected the sources from which they had obtained election news in the past week from a list of 45 news sources (all sources with bias ratings on the website Allsides.com). For each source they selected, they were asked how many hours of election news they obtained from it in the last week. Finally, participants answered two honesty questions. They were asked if they responded randomly or without reading any questions and if they looked up any headlines online. Participants who responded yes to either question were removed from analyses.

Participants completed the study on Wednesday, 11 November 2020, eight days after the election, and four days after the result had been called by all major news sources. These methods allowed us to investigate factors associated with susceptibility to misinformation about election fraud in the days after the election.

## 3. Results

We first conducted correlational analyses. The descriptive statistics and bivariate correlations between measures are presented in Table 1. Political ideology, approval of the outgoing president and conspiracy beliefs were positively correlated with belief in false election news, whereas media trust and cognitive reflection were negatively correlated with it (all *p* < 0.001). Having a college degree was not significantly related to belief in fake news. Many of the predictors were intercorrelated. For example, political ideology and approval of Trump were strongly correlated.

To examine the unique variance in belief in false news explained by each predictor, we conducted a simultaneous multiple linear regression analysis. We first confirmed that the data met assumptions for this analysis. Next, we entered political ideology, approval of the outgoing president, media trust, cognitive reflection, conspiracy beliefs and college education as predictors and belief in false headlines as the outcome. As shown in Table 2, political ideology, approval of Trump and conspiracy beliefs were positively associated with belief in false headlines, whereas cognitive reflection was negatively associated with it. This model explained a significant proportion of variance in belief in false news, *F*(6, 369) = 98.31, *p* < 0.001, *R*^2^ = 0.62 (adjusted *R*^2^ = 0.61). Despite having a significant bivariate correlation with belief in false news, media trust was not significantly related to it in the full model.

Next, we examined how many hours participants claimed to have spent obtaining news about the election and how this related to their belief in false election news. We first separated news sources by their ratings on Allsides, which are as follows: left (e.g., MSNBC), leans left (e.g., CNN), center (e.g., AP), leans right (e.g., Fox News) and right (e.g., Brietbart). The mean hours for each leaning, the total mean hours and the correlations with belief in false election news are presented in Table 3. The more hours of news consumed that were left, leaned left and center were all associated with less belief in false election headlines, whereas the more hours of news consumed that were right and leaned right were both associated with greater belief in false election headlines. In addition, the more total hours participants reported consuming election news, the less they believed in false election headlines.

We also examined belief in true election news. These analyses were not preregistered. Table 1 contains the bivariate correlations between belief in true election news and our individual difference measures. Belief in true news was positively associated with media trust and cognitive reflection; negatively associated with political ideology, approval of the outgoing president and conspiracy beliefs and not significantly associated with having a college degree. Overall, these findings mirrored those of belief in false election headlines, though the strengths of the associations tended to be greater with belief in false news than with belief in true news. Table 3 contains the correlations between belief in true news and the number of hours of news obtained from each political leaning category of news sources. More hours of news consumed that leaned right was associated with less belief in true election headlines, whereas more hours of news consumed from sources that were left, leaned left and center were all associated with greater belief in true election headlines. The number of hours from right news sources was unrelated to belief in true headlines. In addition, the more total hours participants reported consuming election news, the more they believed in true election headlines. These results also mirrored those of belief in false headlines.

## 4. Discussion

Our findings indicated that political conservatism and greater approval of the outgoing president were associated with more belief in false headlines about election fraud. Relatedly, in planning their defense, several rioters involved in the insurrection of the Capitol intend to blame Trump’s promotion of the election fraud conspiracy [12]. Greater susceptibility to conspiracy narratives was also related to belief in false headlines. This relationship has been demonstrated in other contexts, including Chilean political fake news [31]. Poorer cognitive reflection was also associated with belief in false headlines, consistent with studies that used different fake news topics [19]. Additionally, self-reported consumption of less news from left-leaning and center news sources and more news from right-leaning news sources was associated with greater belief in these false headlines. This finding is consistent with the one that showed that more consumption of right-leaning news media was associated with poorer discernment between true and false political headlines [15]. Education was not significantly related to belief in false headlines. In previous studies, education level was associated with greater liking and sharing of false news about climate change [4] and greater discernment between true and false political headlines [19,25], but was not significantly associated with greater belief in COVID-19 false information [32].

Together, the present findings and the observed consequences of believing in false stories of election fraud [10] suggest that interventions are needed to mitigate susceptibility to election misinformation. In other contexts, studies have shown that inoculating people against misinformation can be effective [33]. In these studies, participants play a game in which they learn about misinformation techniques. After playing the game, participants are less susceptible to misinformation [33,34,35]. The benefits associated with these games can last for some time [36]. Information literacy may also reduce susceptibility to misinformation [37], and news literacy messages can decrease the perceived credibility of misinformation [38]. Similar approaches may be helpful in reducing beliefs in false stories of voter fraud. However, some interventions are unsuccessful when headlines are political [18]. It may be that perceptions about election fraud are too deeply entrenched for interventions to succeed.

In addition to interventions designed to combat electoral misinformation, interventions to reduce the allure of conspiracy narratives are necessary. For instance, critical thinking lessons can reduce conspiracy beliefs in college students [39]. Additionally, providing rational arguments against a specific conspiracy narrative can successfully reduce belief in it [40]. Further research is needed to determine the most effective ways to reduce conspiracist ideation, but educational approaches to reducing susceptibility to false news and conspiracy narratives may hold promise.

Crucially, these interventions—whether specifically crafted to reduce false perceptions of electoral fraud or more generally to combat conspiracy narratives—must engage the audience most susceptible to such misinformation. Given that people are more likely to believe information presented by sources they trust [41], it is important that interventions intended to nullify electoral misinformation be deployed in media ecosystems frequented by susceptible individuals. Here, that means that conservative-leaning media advertisements or conservative-targeted social media interventions will be needed. Moreover, simply by being presented in right-leaning media, the interventions are much more likely to reach the people who believe in false news about electoral fraud.

There were limitations in this study. One limitation is that we did not have a representative sample. Certain individuals, particularly those with strong conspiracy beliefs, may be unlikely to participate in online studies. Mechanical Turk samples tend to be more educated and more politically liberal than representative samples [42], and both education and liberal ideology are related to less belief in conspiracy theories [43,44]. Therefore, conspiracy-prone individuals may be underrepresented in the sample. Another limitation is the small number of false headlines about election fraud and the small number of individual difference measures. To minimize the time demands on participants, we selected only the measures we thought were most important. 

Our findings shed light on the qualities predicting susceptibility to a particularly problematic class of fake news, namely, belief in election fraud. This belief has already had deadly consequences, and it continues to permeate a large portion of the electorate and parts of the government itself [45]. Belief in election fraud has also triggered bad-faith election audits, and these audits are harming democracy [46]. Because the misinformation spread by politicians and political pundits is effective in instilling belief among their supporters, it is important to counter with interventions designed to reduce false beliefs and conspiracy ideation, and to target those interventions at conservatives by presenting them in right-leaning media contexts.

## Figures and Tables

**Table 1:** Means, standard deviations, and intercorrelations of belief in fake news and individual difference measures employed in the study.

	M	SD	2.	3.	4.	5.	6.	7.	8.
1. Belief in false news	2.62	1.12	−0.32 *	0.56 *	0.72 *	−0.49 *	−0.22 *	0.50 *	−0.06
2. Belief in true news	4.76	0.88	-	−0.28 *	−0.40 *	0.24 *	0.14 *	−0.26 *	0.10
3. Political ideology	3.54	1.90		-	0.67 *	−0.63 *	−0.11 *	0.23 *	0.05
4. Trump approval	2.03	1.17			-	−0.56 *	−0.15 *	0.33 *	−0.03
5. Media trust	2.94	1.29				-	−0.03	−0.28 *	0.12 *
6. Cognitive reflection	3.75	1.96					-	−19 *	0.14 *
7. Conspiracy beliefs	2.60	1.03						-	−0.17 *
8. College degree	-	-							-

* Correlations are statistically significant (*p* < 0.05).

**Table 2:** Standardized regression coefficients for the associations with belief in false election news.

	β	SE	*p*
Political ideology	0.10	0.05	0.045
Trump approval	0.52	0.05	<0.001
Media trust	−0.07	0.04	0.132
Cognitive reflection	−0.09	0.03	0.010
Conspiracy beliefs	0.27	0.03	<0.001
College degree	0.02	0.03	0.599

**Table 3:** Means, standard deviations and correlations with belief in false election news for the number of hours of election news consumed for each political leaning of news sources.

			Belief in False News		Belief in True News	
	M	SD	r	*p*	r	*p*
Left news hours	1.60	3.69	−0.13	0.009	0.12	0.021
Leans left hours	6.36	8.11	−0.28	<0.001	0.26	<0.001
Center hours	2.44	4.23	−0.15	0.003	0.22	<0.001
Leans right hours	1.47	2.80	0.17	0.001	−0.16	0.001
Right hours	0.74	3.48	0.20	<0.001	0.03	0.608
Total news hours	12.61	15.54	−0.15	0.005	0.20	<0.001

## Data Availability

The data presented in this study are openly available on the Open Science Framework (DOI: 10.17605/OSF.IO/HRCM3).
